# Phase II Study of Biweekly Pemetrexed Plus Irinotecan as Second-Line Therapy for Metastatic Colorectal Cancer

**DOI:** 10.1155/2010/785934

**Published:** 2010-04-08

**Authors:** C. Louvet, T. André, E. Gamelin, M. Hebbar, M. Mabro, M. Bennamoun, H. Rassam, A. de Gramont

**Affiliations:** ^1^Service d'Oncologie, Hôpital Saint-Antoine, 184 rue du Faubourg Saint-Antoine, 75012 Paris, France; ^2^Groupe Hospitalier Pitié Salpétrière, Service d'Hépato-Gastroentérologie, Paris, France; ^3^Centre Paul Papin, Angers, France; ^4^Hôpital Claude-Huriez, Lille, France; ^5^Hôpital Foch, Suresnes, France; ^6^Centre Hospitalier Intercommunal Le Raincy-Montfermeil, Montfermeil, France; ^7^Eli Lilly, France

## Abstract

*Background*. This open-label, single-arm, two-stage, Phase II study investigated the efficacy and safety of bi-weekly pemetrexed combined with irinotecan, in patients with metastatic colorectal cancer (mCRC), after first-line chemotherapy using FOLFOX regimen. *Patients and methods*. Patients received pemetrexed 400 mg/m*²* as a 10-minute intravenous infusion (with vitamin supplementation) followed by irinotecan 180 mg/m*²* as a 90-minute infusion on day 1 of a 14-day cycle, for a maximum of 12 cycles. The primary endpoint was response rate (RR; H_0_ ≤ 5%, H_*a*_ ≥ 20%, *α* = 0.05, power = 90%). Secondary endpoints were duration of response, progression-free survival (PFS), overall survival (OS), and toxicities. *Results*. Partial response was observed in six out of 44 patients enrolled in the study (RR = 13.6%). The median PFS and OS were 4.0 and 13.9 months, respectively. The most common grade 3-4 toxicities were fatigue: 20.5% of patients, neutropenia: 18.6%, diarrhea: 13.6%, elevated transaminases: 9.5%, anemia: 9.3%, and vomiting: 6.8%. *Conclusion*. Pemetrexed plus irinotecan administered every two weeks is an active and well-tolerated regimen in mCRC patients pretreated with FOLFOX regimen. However, this regimen does not seem to provide clinically relevant advantage over historical data of a classical FOLFIRI regimen.

## 1. Introduction

Colorectal cancer accounts for 10% to 15% of all cancers and is the second leading cause of cancer deaths in western countries. Approximately half of all patients develop metastatic disease [1]. In many patients, disease is too advanced for any treatment other than palliative therapy. Efficacy of front-line chemotherapy for the treatment of patients with metastatic colorectal cancer (mCRC) has been improved by the use of combined treatments of irinotecan or oxaliplatin with fluoropyrimidines. The addition of irinotecan to bolus 5-fluorouracil/leucovorin (5-FU/LV) increased median survival in patients with mCRC from 12 to 14.8 months [2]. This rate was increased further by combining irinotecan or oxaliplatin with infusion-based 5-FU/LV however, doublets such as irinotecan plus infusional 5-FU/LV (FOLFIRI) or oxaliplatin plus infusional 5-FU/LV (FOLFOX) prolonged median survival to more than 20 months [3–6]. Both regimens, FOLFOX and FOLFIRI, are recognized as standard first-line therapies for mCRC [7]. However, the 5-year survival rate remains poor (less than 10%).

The clinical benefit of second-line therapy in patients with progressive disease remains unsatisfactory. Few data are available about irinotecan-based chemotherapy in patients previously treated with FOLFOX. The FOLFIRI regimen achieved only 5% to 10% response rate after FOLFOX in heavily pretreated patients, and optimization of irinotecan-based regimens is clearly needed [8, 9]. 

Pemetrexed (Alimta) is a multitargeted antifolate agent that inhibits several key folate-dependent enzymes required for de novo purine and/or pyrimidine biosynthesis, including thymidylate synthase (TS), dihydrofolate reductase (DHFR), and glycinamide ribonucleotide formyltransferase (GARFT) [10]. Pemetrexed has shown broad clinical antitumor activity in patients with colorectal, pancreatic, and breast cancers [11] and has received regulatory approvals for treating patients with malignant mesothelioma and nonsmall cell lung cancer [12]. Pemetrexed 500–600 mg/m² administered every three weeks showed single-agent activity as first-line treatment for advanced CRC in two phase II trials [13, 14]. The objective responses rates were 15% in the American study [14] and 17% in the Canadian study [13] and the median overall survival times were 16.2 and 15.1 months, respectively. The major toxicities of pemetrexed are myelosuppression, skin rash, and mucositis, with neutropenia being the primary dose-limiting toxicity [15]. An elevated level of plasma homocysteine was found to be a significant risk factor for treatment-related toxicities [16]. Supplementation with vitamin B_12_ and folic acid has been shown to lower plasma homocysteine level and improve the toxicity of pemetrexed. 

The distinct mechanisms of action and patterns of resistance displayed by pemetrexed and irinotecan make them attractive agents for combination therapy in mCRC patients. The combination of pemetrexed and irinotecan, administered every three weeks, was shown to be feasible in phase I studies [17, 18]. In the phase I/II study reported by Hochster et al., pemetrexed 500 mg/m² followed by irinotecan 300 mg/m² on day 1, every 21 days, induced an objective response rate of 11% in 35 patients previously treated with 5-FU-based chemotherapy for advanced disease [17]. 

This multicenter, nonrandomized, open-label, single-arm phase II study was initiated to evaluate the efficacy and safety of bi-weekly pemetrexed plus irinotecan, after failure to the FOLFOX regimen in patients with mCRC.

## 2. Patients and Methods

### 2.1. Patients

Male and female patients of at least 18 years of age, with histologically or cytologically confirmed mCRC progressive after first-line chemotherapy with a single FOLFOX regimen (5-FU, leucovorin, and oxaliplatin), were eligible for the study. Further inclusion criteria included the following: ECOG (Eastern Cooperative Oncology Group) performance status of 0, 1, or 2; life expectancy of at least 12 weeks; at least one site of measurable metastatic lesion; no prior radiation therapy to bone marrow exceeding 25% of hematopoietic reserves and adequate hematologic (absolute neutrophil count [ANC] ≥1.5 ×10^9^/L, platelet count ≥100 × 10^9^/L, and hemoglobin ≥9 g/dL), hepatic (serum bilirubin ≤ 1.5 times the upper limit of normal (ULN); alkaline phosphatase (ALP), aspartate transaminase (ASAT) and alanine transaminase (ALAT) ≤ 3.0× ULN or ≤5× ULN in case of hepatic metastases) and renal (calculated creatinine clearance (CrCl) ≥ 45 mL/min) functions. Exclusion criteria included pregnant or breast-feeding women, prior treatment with irinotecan (except adjuvant treatment administered more than 6 months before study entry), documented brain metastases not amenable to surgery or unstable after radiation, unwillingness or inability to take vitamin B_12_ or folic acid, and history of weight loss (≥10%) over the previous 6 weeks before study entry. 

The study was conducted in accordance with the Declaration of Helsinki and the guidelines of Good Clinical Practice. The institutional review boards of participating centers approved the study, and patients gave written informed consent before enrollment.

### 2.2. Treatment Plan

All patients received 400 mg/m² pemetrexed (Alimta, Eli Lilly and Company, Indianapolis, Indiana) as a 10-minute intravenous (i.v.) infusion followed by 180 mg/m² irinotecan as a 90-minute i.v. infusion on day 1 of a 14-day cycle. Cycles were repeated until disease progression, unacceptable toxicity, and investigator or patient decision, with a maximum of 12 cycles. Folic acid oral supplementation of 350–600 *μ*g or equivalent was given daily beginning 1 to 2 weeks prior to Day 1 of Cycle 1 and continuing daily until 3 weeks after the last pemetrexed dose. Vitamin B_12_ 1000 *μ*g was injected intramuscularly 1 to 2 weeks prior to the first pemetrexed dose then every 9 weeks until 3 weeks after the final dose. Dexamethasone (4 mg or equivalent) was administered orally twice daily on the day before, the day of, and the day after each dose of pemetrexed.

Dose adjustments or delays during the study were based on hematologic and/or nonhematologic toxicities in the preceding cycle, graded according to the National Cancer Institute-Common Terminology Criteria for Adverse Events (NCI-CTCAE, version 3.0, 2003). Patients were required to have an ANC of at least 1.5 × 10^9^/L and a platelet count of at least 75 × 10^9^/L before treatment on the first day of each cycle. The doses of both drugs were delayed (until resolution or return to baseline) and modified for either ANC <1.0 × 10^9^/L and a platelet count of ≥50 × 10^9^/L (25% dose reduction) or a platelet count of <50 × 10^9^/L (50% reduction). Similarly, treatment was delayed for insufficient folic acid or vitamin B_12_ supplementation, grade 3/4 nonhematologic toxicities (except for grade 3 transaminase elevation, nausea, vomiting, and alopecia), or calculated CrCl <45 mL/min. When nonhematologic toxicities resolved, therapy resumed at 75% of the previous level for grade 3 diarrhea (only the dose of irinotecan was reduced for these patients), grade 3 or 4 vomiting despite antiemetic premedication, and any other grade 3/4 nonhematologic toxicity deemed appropriate. Therapy resumed at 50% of the previous level for grade 3 or 4 mucositis or grade 4 diarrhea (irinotecan only). Dose reescalation was not allowed. Any patient requiring a third dose reduction was discontinued from the study. 

Concomitant treatments included atropine for cholinergic symptoms (preventive treatment was given if severe acute cholinergic symptoms were experienced during a previous cycle) and loperamide as a curative treatment. Premedication with an antiemetic regimen including an HT3 antagonist was recommended.

### 2.3. Study Assessments

Disease status was assessed at baseline by a complete medical history and physical examination with abdominal computed tomography (CT) scans and chest X-ray, performance status (PS) measurement and complete blood chemistry and hematology. During treatment, physical examinations and PS assessments, hematology and serum chemistry were carried out before each cycle. CT-scans and CEA levels were performed every 4 cycles to document response, then 30 days after the last dose of study drugs and every 3 months for two years.

### 2.4. Evaluation of Response and Toxicity

Objective tumor response was rated using Response Evaluation Criteria in Solid Tumors (RECIST) guidelines [19]. Complete response (CR) was defined as the disappearance of all clinical and radiological evidence of target lesions; partial response (PR) as a ≥ 30% decrease in the overall sum of the longest diameter of the target lesion(s) taking as reference the baseline sum, and progressive disease (PD) as a ≥ 20% increase in the overall sum of the longest diameter of the target lesion(s) taking as reference the smallest sum recorded since the treatment started. In case of PR or CR, a second assessment was required 4 weeks later for confirmation of response. The duration of response was defined as the time from the first objective status assessment of CR or PR to the first time of progression or death due to any cause. The duration of response was censored at the date of the last follow-up visit for patients who neither had progression nor died due to any cause. Progression-free survival (PFS) was defined as the time from study enrollment to the first observation of disease progression or death due to any cause. PFS was censored at the date of the last follow-up visit for patients who neither had progression nor died due to any cause. Overall survival was the time from study enrollment to time of death from any cause. Overall survival was censored at the date of the last follow-up visit for patients who had been still alive or lost to follow-up. 

Toxicity was recorded before each cycle and graded according to the NCI-CTCAE (version 3.0, 2003).

### 2.5. Statistical Analysis

Forty-five patients had to be enrolled in this single-arm, two-stage, sequential phase II study with the possibility of stopping the study early because of lack of efficacy assuming a 10% drop-out rate [20]. A total of 20 evaluable patients were to be entered in the first stage. If at least one response was observed in the first 20 patients, 20 additional patients evaluable for response were to be accrued (total of 40 evaluable patients). If fewer than 5 of 40 patients responded to therapy, the regimen had to be deemed not worthy of any further investigation in this patient population, unless clinical considerations suggest otherwise. If responses were seen in greater than or equal to 5 of 40 patients, the regimen was to be recommended for further study. If the true response rate was 5%, the probability that this procedure would conclude that the regimen was worthy of further study was  0.05 (significance). If the true response rate was 20%, this procedure had power of 90% to conclude that the regimen was worthy of further study.

The primary endpoint was the best overall objective response rate (complete plus partial responses) and its exact 95% confidence interval (CI) based on F distribution method (Leemis and Trivedi, 1996) was presented. All patients who had received at least one complete cycle were included in the primary outcome analysis. Secondary outcomes were PFS, overall survival, and incidence of adverse events. The distribution of time-to-event endpoints was estimated using the Kaplan-Meier method (Kaplan and Meier, 1958), 95% CI for the quartiles was based on the sign test (Brookmeyer and Crowley, 1982). For toxicity analysis, the worst grade for each patient was used.

## 3. Results

Forty-six patients were entered in the study by six French centers between 06/2004 and 01/2006. Two patients were withdrawn before the beginning of the study treatment due to unmet protocol entry criteria and patient's decision (44 patients included). Forty-four patients received at least one complete cycle of chemotherapy.

### 3.1. Patient Characteristics

The baseline characteristics of the patients (*N* = 44) are summarized in Table 1. The majority of the patients were male (70.5%) and the median age was 63 years (range: 33 to 81 years). The median time from initial diagnosis to inclusion was 17 months. The majority of patients (75.0%) had two or more synchronous metastatic sites with liver (70.5%) and lung (40.9%) as the most common sites of metastases. Liver-only disease was present in 11 patients. The majority of patients (86.4%) underwent prior surgery while 29.5% had received adjuvant treatment, and all had previously been treated with a FOLFOX (*n* = 41) or a XELOX (*n* = 3) regimen for metastatic disease.

### 3.2. Treatment Delivery

The median number of treatment cycles administered was 4.5 (range: 1 to 14 cycles). The median relative dose intensities (RDI—actual/planned doses) were 96.5% for pemetrexed (range: 47.9% to 102.8%) and 95.2% for irinotecan (range: 61.8% to 102.5%). The doses of pemetrexed and irinotecan had to be reduced due to the occurrence of an adverse event for 18% and 23% of patients, respectively. The cycles were delayed for 66% of patients, mainly due to scheduling conflict. Seven patients (15.9%) completed the study protocol and 37 patients discontinued the study early due to lack of efficacy (*n* = 21), patient's decision (*n* = 6), physician's decision (*n* = 4), adverse event (*n* = 4), or death (*n* = 2).

### 3.3. Efficacy

**Figure 1:**
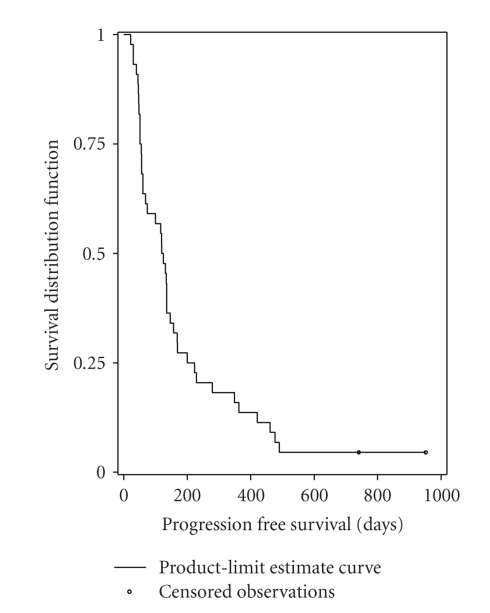
Kaplan-Meier analysis of progression-free survival (*N* = 44).

**Figure 2:**
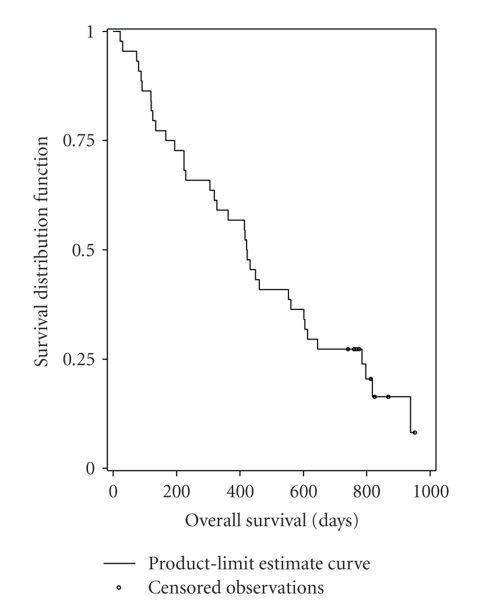
Kaplan-Meier analysis of overall survival (*N* = 44).

One partial response was observed in the first 20 patients, and thus it allowed to continue enrollments up to a total of 44 patients in accordance with the protocol. In the population of patients receiving at least one complete cycle of chemotherapy (*N* = 44), the best overall response rate was 13.6% (95% CI, 5.2% to 27.4%). Table 2 provides the best confirmed response information. There were 6 partial responses (13.6%), 18 patients (40.9%) had stable disease, 15 patients (34.1%) had progressive disease, and response was unknown for 5 patients (11.4%). The median duration of responses was 7.8 months (95% CI, 3.5 to 11.6 months). The median PFS was 4.0 months (95% CI, 2.0 to 4.8 months) (Figure 1). The median overall survival was 13.9 months (95% CI, 10.0 to 19.8 months) (Figure 2). Thirty-two patients received third-line chemotherapy.

### 3.4. Safety

Table 3 summarizes the grade 3 or 4 toxicities occurring during the study. The most common grade 3 or 4 adverse events possibly related to study drugs were fatigue (20.5% of patients), neutropenia (18.6%), diarrhea (13.6%), elevated ALAT (9.5%), anemia (9.3%), and vomiting (6.8%). Eight patients (18.2%) experienced at least one serious adverse event (SAE) related to study drugs with anemia, diarrhea, vomiting, and dehydration as the most common SAE. Four patients (9.1%) experienced an AE leading to study discontinuation. No toxic death was reported during the study period.

## 4. Discussion

This open, nonrandomized, multicenter, single-arm phase II study assessed the efficacy and safety of pemetrexed plus irinotecan combination as second-line treatment of patients with metastatic colorectal cancer. The combination of pemetrexed plus irinotecan administered every two weeks with vitamin supplementation is an effective and acceptably tolerated regimen in mCRC patients previously treated with FOLFOX. The objective response rate of the combination was 14% and stable disease was reported in 41% of patients. The median PFS and overall survival were 4.0 months and 13.9 months, respectively. These results are close to those reported with pemetrexed plus irinotecan administered every three weeks in patients with advanced CRC previously treated with 5-FU-based chemotherapy [17]. In 35 patients treated with pemetrexed 500 mg/m² followed by irinotecan 300 mg/m² with vitamin supplementation, the objective response rate was 11.4% and the median time to progression and overall survival was 3.7 and 8.1 months, respectively. Usually, a second-line irinotecan-based combination is indicated for mCRC patients after FOLFOX first-line failure. The efficacy of pemetrexed plus irinotecan in the present study seems to be comparable to historical data for FOLFIRI regimens. André et al. reported an overall response rate of 6% and a median survival of 9.9 months in 33 mCRC patients treated by FOLFIRI as third-line therapy [8]. Tournigand et al. demonstrated that FOLFIRI had a low response rate when given after first-line FOLFOX (4%) [9]. FOLFIRI-2 regimen (leucovorin, fluorouracil, irinotecan, and hydroxyurea) induced an objective response rate of 17%, a median PFS of 4.1 months, and a median survival of 9.7 months in 29 patients refractory to 5-FU and oxaliplatin [21]. FOLFIRI-3, in which irinotecan is administered as two infusions (half dose before 5-FU and half dose at the end of the 5-FU infusion), induced a response rate of 23% in 65 mCRC patients pretreated with FOLFOX, a median progression-free survival of 4.7 months, and a median survival of 10.5 months [22]. 

The choice of a second-line therapy in mCRC is now more and more complex. It has to be defined for each patient based on the previous received treatments (adjuvant as well as first-line metastatic), the available active drugs in CRC (conventional chemotherapies as well as targeted therapies), and some genomic properties of the tumor. Actually, anti-VEGF bevacizumab and anti-EGFr cetuximab or panitumumab become available in the treatment of mCRC and could also be indicated as second-line treatments in combination with conventional chemotherapy [23, 24]. To date, anti-VEGF drugs administration is not limited by validated genomic alteration, while anti-EGFr use should be restricted to tumors with a wild-type KRAS phenotype. To our knowledge, no data concerning combination of pemetrexed with targeted drugs in mCRC is available. Interestingly, activity of pemetrexed was demonstrated as related to polymorphism of thymidylate synthase (TS) and methylenetetrahydrofolate reductase (MTHFR) in nonsmall cell lung cancer [25]. Such impact of TS and MTHFR polymorphisms deserves to be analyzed in colon cancer and may help in the definition of subgroups of patients who could better benefit from pemetrexed administration in mCRC. 

The tolerance profile of every two-week pemetrexed plus irinotecan combination was acceptable and manageable in mCRC patients previously treated with FOLFOX. Myelosuppression, general disorders, and gastrointestinal disorders were the most common toxicities resulting from the combination therapy. The most common grade 3-4 toxicities were fatigue (20%), neutropenia (19%), diarrhea (14%), elevated ALAT (9.5%), anemia (9%), and vomiting (7%). Grade 4 toxicity was infrequent and only one patient experienced febrile neutropenia. The toxicity profile of the same combination administered every three weeks as second-line therapy was similar except for the incidence of fatigue, which was higher in the every-two week schedule [17]. However, the grade 3-4 toxicities (mainly fatigue and diarrhea) led to early treatment discontinuation in 10 patients (23%). This, combined with a usual short PFS in second-line mCRC patients, explained the low 4.5 median number of administered cycles.

In conclusion, pemetrexed plus irinotecan administered every two weeks is an active and well-tolerated regimen in mCRC patients pretreated with FOLFOX regimen. However, based on historical data, this regimen seems not to provide clinically relevant advantage over combinations of 5-FU and irinotecan in second-line mCRC treatment. This was also the case in first-line therapy, where the every-three-week pemetrexed plus irinotecan regimen (ALIRI) did not improve upon the efficacy and safety observed with FOLFIRI [26]. These data do not incite to perform randomized studies comparing pemetrexed-irinotecan to 5-FU-irinotecan. However, this combination could be of interest in patients who experienced severe toxicities when treated with fluoropyrimidines, or in patients with fluoropyrimidines and/or targeted drugs contraindication. Additional data concerning combinations of pemetrexed and biologics and concerning a possible selection of patients who could better benefit from pemetrexed, based on genomic properties of the tumor, are needed.

## Figures and Tables

**Table 1:** Patient characteristics (*N* = 44).

			*N* (%)
Age, years	Median	63.0	
	Range	33–81	
Sex	Male		31 (70.5%)
	Female		13 (29.5%)
ECOG performance status	0		24 (54.5%)
	1		15 (34.1%)
	2		5 (11.4%)
Primary site	Colon		22 (50.0%)
	Rectum		20 (45.5%)
	Colorectal		2 (4.5%)
Metastatic sites	Liver		31 (70.5%)
	Lung		18 (40.9%)
	Lymph nodes		8 (18.2%)
	Other (mediastinum, pelvis, perineum)		4 (9.1%)
	Peritoneum		2 (4.5%)
Number of target lesions	1		11 (25.0%)
	2		12 (27.3%)
	3		10 (22.7%)
	4 or more		11 (25.0%)
Prior therapy	Surgery		38 (86.4%)
	Radiotherapy		11 (25.0%)
	Chemotherapy		
	Metastatic		44 (100.0%)
Time from initial diagnosis to inclusion, months	Median	17.0	
	Range	2.8–81.1	

Abbreviation: ECOG: Eastern Cooperative Oncology Group.

**Table 2:** Best confirmed response in patients who received at least one complete cycle of chemotherapy (*N* = 44).

Best tumor response	Number of patients	%
Partial response	6	13.6
Stable disease	18	40.9
Progressive disease	15	34.1
Unknown	5	11.4

**Table 3:** Number of patients who experienced a grade 3 or 4 toxicity.

NCI-CTCAE grade 3-4 toxicity	Grade 3	Grade 4
Hematological toxicity		
Anemia	3 (7.0%)	1 (2.3%)
Leucopenia	1 (2.3%)	2 (4.7%)
Neutropenia	5 (11.6%)	3 (7.0%)
Thrombocytopenia	1 (2.3%)	0 (0.0%)

Nonhematological toxicity		
Fatigue	9 (20.5%)	0 (0.0%)
Nausea	2 (4.5%)	0 (0.0%)
Diarrhea	5 (11.4%)	1 (2.3%)
Vomiting	3 (6.8%)	0 (0.0%)
Anorexia	2 (4.5%)	0 (0.0%)
Mucositis/stomatitis	2 (4.5%)	0 (0.0%)
Dehydration	2 (4.5%)	0 (0.0%)
Febrile neutropenia	1 (2.3%)	0 (0.0%)
Infection	0 (0.0%)	1 (2.3%)
Rash	1 (2.3%)	0 (0.0%)
Biochemistry		
ALAT increase	4 (9.5%)	0 (0.0%)
Hyperbilirubinemia	2 (4.8%)	0 (0.0%)
Hypokalemia	2 (4.8%)	0 (0.0%)
Alkaline phosphatase	1 (2.4%)	0 (0.0%)
Hyponatremia	1 (2.4%)	0 (0.0%)

Abbreviations: NCI-CTCAE: National Cancer Institute-Common Terminology Criteria for Adverse Events; ALAT: alanine aminotransferase.
